# Dislocation of the External Semilunar Cartilage

**Published:** 1843-12

**Authors:** Caleb Taylor


*Dislocation of the External Semilunar Cartilage.* By Caleb Taylor.—On the morning of the 17th instant I was summoned to Thomas Mills, ætat. 30, a well-proportioned man, resident in this town. He stated that whilst in the act of "cutting his corns," with his foot and leg somewhat twisted inwards, he felt as though something had given way in the knee, and immediately found that he had no longer the power of extending the limb, every attempt at so doing producing severe pain in the joint. He told me that the same accident had occurred to him twice before; the first time in the country, when two surgeons were with him for several hours before the limb was made to resume its normal condition. It again occurred in London, where he applied at St. Thomas's Hospital, and he states that the late Mr. Tyrrell and several others were a long time trying to "put it right," but failed; the plan adopted was by making very powerful pressure over the patella, which caused him severe suffering, and produced much swelling. He was directed to go home, leech freely, apply cold lotions, and keep in bed. On the third day, whilst making a sudden movement in bed, he suddenly recovered the power of straightening the limb or placing it in any position without pain. He felt that "all was right," and resumed his occupation.

I found the leg flexed, and any attempt at straightening produced pain, referred to the situation of the external semilunar cartilage. I fancied (it might be fancy) that I could see a slight elevation of the supra-incumbent tissues, and supposed it to be a partial dislocation of that cartilage. I immediately made extension at the ankle, at the same time adducted the tibia, making the knee of the opposite side a fulcrum, the tendency of which would be to separate the external part of the articulating surfaces of the tibia and femur; his wife, at the same time, was directed to make firm pressure over the cartilage. After keeping up extension for about ten minutes, the parts resumed their normal condition, and, much to his delight, the patient found he could walk with ease, and, in spite of remonstrance, immediately returned to his occupation. The only after treatment was that of directing him to wear a bandage over the knee.—*Lond. Lancet*, Sept., 1843.

